# Dental Implants

**DOI:** 10.1155/2013/838565

**Published:** 2013-02-07

**Authors:** Carlos Nelson Elias, Paulo G. Coelho

**Affiliations:** ^1^Department of Mechanical Engineering and Materials Science, Military Institute of Engineering, Rio de Janeiro, RJ, Brazil; ^2^Department of Biomaterials and Biomimetics, College of Dentistry, New York University, New York, NY 10010, USA

Osseointegration is a major factor influencing the success of dental implants. To achieve rapid and strong durable osseointegration, biomaterial researchers have investigated various surface treatment methods for titanium dental implants. Current dental implant research has studied the interaction between bone and implant surface in order to understand the osseointegration process. This special dental implant issue addresses the role of titanium dental implant surface treatment, chemical or topographic modification, and cells interactions. All these parameters can affect bone healing, promote accelerated osteogenesis, and increase bone-implant contact and bond strength.

The themes include a relevant interest about the properties of dental implant surfaces, analysis of molecular mechanisms which occur at the interface between bone and dental implant, evaluation of the biological behavior of the host's tissues at the interface with the implant, and considerations of implant micro- and nanotopography and its superficial chemical structure. Researchers present their results of investigation of various surface treatment methods for dental titanium implants, show the biological basis for successful implant therapy, and analyzed the effect of plasma fibronectin.

In F. P. S. Guastaldi et al.'s paper, the authors propose a novel plasma treatment for dental implant surfaces. Their results suggested that even after 30 days of plasma treatment, the biological responses were better than those of nontreated surfaces.

In M. Monjo et al.'s paper, the authors compared the cytotoxicity, cell morphology and proliferation, alkaline phosphatase activity, gene expression, and the release of a wide array of osteoblast markers of two commercial titanium (Ti) surfaces (OsseoSpeed and TiOblast). They observed changes in cell shape and BMP-2 secretion after 2 days between the surfaces, and this was followed by increased IGF-I, BSP, osterix gene expression and mineralization after 14 days.

In C. N. Elias et al's paper, the authors research the effects of dental implant surface treatment with fluoride and fibronectin adsorption on the adhesion of osteoblasts. They investigated the biofunctionalization of titanium surfaces and examine their effects on the interaction with osteoblastic cells. They observed that the association indexes of osteoblastic cells in fibronectin-treated samples were significantly higher than those in samples without fibronectin. The radioactivity values suggested that fibronectin incorporation is an important determinant of the *in vitro *cytocompatibility of the surfaces. They concluded that the preparation of bioactive titanium surfaces via fluoride and FN retention proved to be a useful treatment to optimize and to accelerate the osseointegration process of dental implants.

In C. Y. Guo et al.'s paper, the authors focus on surface charge modification on the surface of titanium dental implants. They make an overview on both theoretical explanations on how surface charge affects the implants' osseointegration, as well as a potential surface charge modification method. Additionally, they discuss the insights into the important factors affecting the effectiveness of surface charge modification methods and point out several interesting directions for future investigations on this topic.

In H. O. Schwartz-Filho et al.'s study, they observe the morphological and molecular effect of laminin-1 doping to nanostructured implant surfaces in a rabbit model. They concluded that the protein-doped surface showed higher gene expression of typical genes involved in the osseointegration cascade than that of the control surface. They observed that the osseointegration cascade begins immediately after the implant is placed in the bone, where the blood contiguously interacts with the implant surface.

We hope that this special issue would attract a major attention of the research community. We would like to express our appreciation to all the authors, reviewers, and the Editor-in-Chief Dr. Mohamed Abdel Razek for the great support that made this special issue possible.

*Carlos Nelson Elias* *Carlos Nelson Elias* *Paulo G. Coelho* *Paulo G. Coelho*

